# Updated range map of an endangered salamander and congeneric competitor reveals different niche preferences

**DOI:** 10.1002/ece3.11262

**Published:** 2024-05-20

**Authors:** Jo A. Werba, David A. W. Miller, Adrianne B. Brand, Evan H. Campbell Grant

**Affiliations:** ^1^ U.S. Geological Survey, Eastern Ecological Science Center (Patuxent Wildlife Research Center) SO Conte Anadromous Fish Research Laboratory Turners Falls Massachusetts USA; ^2^ Department of Ecosystem Science and Management Pennsylvania State University University Park Pennsylvania USA

**Keywords:** data integration model, sample design, species distribution model, uncertainty

## Abstract

Estimating distributions for cryptic and highly range‐restricted species induces unique challenges for species distribution modeling. In particular, bioclimatic covariates that are typically used to model species ranges at regional and continental scales may not show strong variation at scales of 100s and 10s of meters. This limits both the likelihood and usefulness of correlated occurrence to data typically used in distribution models. Here, we present analyses of species distributions, at 100 × 100 m resolution, for a highly range restricted salamander species (Shenandoah salamander, *Plethodon shenandoah*) and a closely related congener (red‐backed salamander, *Plethodon cinereus*). We combined data across multiple survey types, account for seasonal variation in availability of our target species, and control for repeated surveys at locations– all typical challenges in range‐scale monitoring datasets. We fit distribution models using generalized additive models that account for spatial covariates as well as unexplained spatial variation and spatial uncertainty. Our model accommodates different survey protocols using offsets and incorporates temporal variation in detection and availability resulting from survey‐specific variation in temperature and precipitation. Our spatial random effect was crucial in identifying small‐scale differences in the occurrence of each species and provides cell‐specific estimates of uncertainty in the density of salamanders across the range. Counts of both species were seen to increase in the 3 days following a precipitation event. However, *P. cinereus* were observed even in extremely wet conditions, while surface activity of *P. shenandoah* was associated with a more narrow range. Our results demonstrate how a flexible analytical approach improves estimates of both distribution and uncertainty, and identify key abiotic relationships, even at small spatial scales and when scales of empirical data are mismatched. While our approach is especially valuable for species with small ranges, controlling for spatial autocorrelation, estimating spatial uncertainty, and incorporating survey‐specific information in estimates can improve the reliability of distribution models in general.

## Graphical abstract

Bioclimatic covariates that are usually used to model species ranges often won't show strong variation at scales of 100s and 10s of meters creating a challenge for creating range maps for species with small distributions. We demonstrate how a flexible analytical approach can improves estimates of both distribution and uncertainty and identify key abiotic relationships at smaller scales. We present analyses of species distributions, at 100 × 100 m resolution, for a highly range restricted salamander species (Shenandoah salamander) and a closely related congener (red‐backed salamander). While our approach is especially valuable for species with small ranges, controlling for spatial autocorrelation, estimating spatial uncertainty, and incorporating survey‐specific information in estimates can improve the reliability of most distribution models.

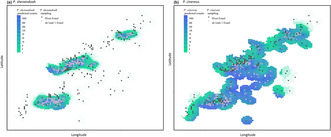

## 1 INTRODUCTION

Range maps depicting the area of a species' expected occurrence form the basis for many conservation and prioritization plans (e.g., Srivathsa et al., 2020). Range maps are useful for finding areas that were historically occupied (Rutrough et al., 2019), highlighting areas with potential habitat to target for conservation actions (e.g., Clancy et al., 2020; Crawford et al., 2020), and for scenario modeling (Harfoot et al., 2021). Additionally, range maps are often needed to guide assessments of potential harm to rare, threatened, and endangered species (Bennun et al., 2018). Given the pace of environmental change, improving and updating range maps may assist conservation decision making (e.g., O'Connor et al., 2019).

Range maps may be constructed from multiple empirical data sources, including field detection/nondetection surveys (e.g., Shen et al., 2021), presence‐only or presence/absence data (e.g., Ficetola et al., 2014), and expert opinion (e.g., Aizpurua et al., 2015; O'Leary et al., 2009). Each of these data streams has associated uncertainty, which is not always accounted for (Fletcher Jr et al., 2019). As a result, many range maps include areas that are actually unsuitable for the species (Hurlbert & White, 2005). For example, a study by Ramesh et al. (2017) found that range maps of 17 of 18 endemic birds misrepresented their actual range. This greatly reduces the ability to use maps to prioritize conservation actions, forecast the extinction risk of a species, or understand which species may be most at risk from land use and environmental change. In particular, range edges are likely to have more uncertainty than the central portion of the range, so mapping edges requires more robust treatment and characterization of the uncertainty.

Uncertainty in range maps comes from a variety of sources, such as spatial sampling and mapping field occurrences (e.g., Lissovsky & Dudov, 2021); modeling (e.g., Hastie & Fithian, 2013; Konowalik & Nosol, 2021; Morales et al., 2017) and model assessment choices (e.g., Konowalik & Nosol, 2021; Lyons et al., 2018); and when forecasting changes in response to future scenarios (including climate futures, e.g., Morales‐Barbero & Vega‐Álvarez, 2019). Thus, representing uncertainty in predictions spatially is critical. Here, we explore how field sampling choices and model specifications affect both the predictions of occurrence and the uncertainty in these predictions for a small‐range, federally endangered montane salamander and a widespread congeneric which are present in the same habitats.

*Plethodon* salamanders are lungless terrestrial salamanders that have no aquatic stage and typically small home ranges. They are a widespread group with highly variable range sizes among species from much of the north central and eastern United States, examples include the widespread *P. cinereus* (Conant & Collins, 1998), to local endemic species such as *P. sherando* (Bayer et al., 2012) and *P. neomexicanus* (Bartlow et al., 2022). Distributions of plethodontid salamanders are presumed to be primarily driven by precipitation (Nottingham & Pelletier, 2021) and temperature (Newman & Austin, 2015) along with microhabitat preferences, all of which are expected to change as a result of global climate change (Farallo & Miles, 2016). Cryptic behaviors and large periods of time spent beneath the soil surface make many species hard to detect even when they are present (Halstead et al., 2022; Kellner & Swihart, 2014).

The Shenandoah salamander (*Plethodon shenandoah*) has a presumed range size of less than 6km^2^ (Grant et al., 2014), but it is unclear how accurate current range maps are because they are derived from data from limited sampling, coupled with hand‐drawn maps based on field‐observed potential suitable habitat as mapped in in the early 1990s by National Park Service staff (USFWS, 1994). Previous work suggested declines in *P. shenandoah* populations due to competition with the red‐backed salamander (*P. cinereus*) would be expected to reduce the range of the species (Jaeger, 1980), though more recent work fails to find a strong effect of competition between the species (Amburgey et al., 2019; Dallalio et al., 2017). The U.S. National Park Service, the State of Virginia, and the U.S. Fish and Wildlife Service are responsible for protection of the species and require accurate information on the extent of occurrence and the probability of occurrence in specific locations where Park maintenance activities may occur (Grant et al., 2014; USFWS, 1994).

Here we present an updated range map of *P. shenandoah*, and for *P. cinereus* overlapping range. Our goal was to improve estimates for the range of the *P. shenandoah*, combining data collected across different survey types. By combining data sources, we sought to estimate current range boundaries and identify spatial gaps in data that lead to uncertainties in range delineation. Our approach addressed four common challenges to distribution modeling in general, and for range‐restricted and cryptic species specifically. These were: (1) the need to combine data sources collected under different study designs and under different environmental conditions, (2) uncertainty associated with cryptic and hard to detect species, (3) making predictions at scales smaller than typically quantified by bioclimatic datasets, and (4) quantifying small‐scale local uncertainty in range boundary estimates. We accomplished this by building a fine‐scale data integration model with both environmental covariates and spatial random‐effects to quantify variation in salamander densities across the geographic area surveyed. We present estimates of relative density and quantified measures of range uncertainty to update our understanding of the current range and identify priority locations for additional sampling effort where uncertainty remains. In addition, we explore the effects of sampling intensity and design on our results, and describe similarities and differences between the two species in their relationships to environmental predictors.

## 2 METHODS

### 2.1 Field collection (survey methods)

We combined survey data from multiple studies of *P. shenandoah*, each of which had unique sampling designs and research objectives. We included all known systematic survey data sets that have been collected in a 15 year window (Amburgey et al., 2019, 2020; Dallalio et al., 2017; Grant et al., 2024; Sevin, 2014). Once combined, our data set included 11,430 unique surveys for 583 sites collected over 15 years (2007–2022). Sampling sites were distributed across 43.5 km^2^ of high‐elevation habitat in Shenandoah National Park, Virginia, U.S.A. that included both the historically estimated range and surrounding areas. Sampling sites were selected according to the unique needs of each individual project, and all were selected based on some form of probabilistic sampling. All surveys included systematic sampling by lifting cover objects within some defined area. The sampling included transects and square plots, and either one or two transects were surveyed on each sampling occasion. Placement of survey sites followed one of three sampling designs, either being randomly selected across the area, stratified or weighted sampling by historical range areas, or linear transects of sampling points that either crossed known range boundaries or followed elevational gradients. Frequency of visits varied over the course of the study period such that sites were surveyed between 0 and 17 times annually. This high level of temporal replication (in addition to the spatial replication among sites) provided important information to our efforts as most individual animals in a population at any point in time are expected to be unavailable for detection (i.e., not on the surface or under cover objects when sampling occurred). The temporal replication allowed us to reduce uncertainty, due to missed detection, and also allowed us to quantify how environmental conditions during a survey event affected the relative availability of animals. Examples of the study objectives for each of the past sampling efforts included efforts to: refine the known range and sample habitats of previously unknown status (Sevin, 2014), define the lower elevation limits (Grant et al., 2018) and lateral range boundaries (Amburgey et al., 2019), collect individuals for captive experiments (Dallalio et al., 2017), examine co‐occurrence zones of *P. shenandoah* and *P. cinereus* (Amburgey et al., 2020; Grant et al., 2024), and estimate annual occupancy of *P. shenandoah* across the high‐elevation habitat (Grant et al., 2023). Surveys were conducted primarily during the day by searching available cover within a defined survey area, where the total area searched and time of year varied by project and survey occasion (Appendix: Tables A1 and A2 for full information on surveys. Raw data and covariate correlations can be found in Figures S10–S12). It is important to note that the survey areas are small relative to the grid cell size used in our model and the model output which is in number of animals per hectare.

In addition to the survey‐specific count, effort, and location data, we extracted site‐level covariates from available GIS databases. For each surveyed site, we estimated elevation, aspect, and slope from a 15 m Digital Elevation Model (DEM). These measures were also used to generate two derived measures of local environmental conditions: a Heat Load Index (HLI), and an Integrated Moisture Index (IMI). These metrics quantify relative measure of variation in solar radiation and relative available moisture, respectively. Each was calculated from the DEM using the ArcMap toolbox (ESRI, 2015), and weather data (precipitation and temperature) were taken from the PRISM data set (PRISM Climate Group, Oregon State University, https://prism.oregonstate.edu). We used daily PRISM data to quantify the average temperature for the 10 previous days, and precipitation over the three previous days for every survey visit. These measures were chosen as proxies to quantify temporal variation in local soil temperatures and moisture, which are important for salamander detection (Muñoz et al., 2016).

### 2.2 Model

The general structure of our species distribution estimator was to fit a zero‐inflated Poisson model, allowing us to account for large number of zero detections while providing a flexible framework to incorporate environmental covariates, random effects, and spatial correlation. This is an appropriate model (Feng, 2021) since zeros in our data can be because of both true absence (structural zeros) or low detection probabilities (sampling zeros). This model also allowed us to fully incorporate our count information from surveys, rather than simplifying data to only include presence‐absence information. Studies for these and similar species suggest that even under ideal conditions, <15% of the total salamander population will be observed during surveys (with the rest of the population either below the surface and unavailable to be observed or going undetected; Bailey et al., 2004a, 2004b, Sanchez et al., 2020). Thus, observed densities will be lower than actual densities, but should scale proportionally.

Following equations from Feng (2021), we estimated the probability of observing a given count *y* during survey *i* to be:

$$P\left(Y_{i}=y_{i}\right)=\left\{\begin{array}{c} \pi_{i}+\left(1-\pi_{i}\right)e^{-\mu_{i}}\;\text{if}\;y_{i}=0\\ \left(1-\pi_{i}\right)\frac{e^{-\mu_{i}}{\mu_{i}}^{y_{i}}}{y_{i}!}\;\text{if}\;y_{i}>0 \end{array}\right.$$

To better account for spatial variation not addressed by our environmental covariates and non‐linear environmental relationships, *π* and *μ* were estimated using a using a spatial generalized additive model (GAM). Where π is probability of a structural zero (*y*_*i*_ = 0) and μ is the mean of the Poisson distribution. GAMs are a good choice to understand environmental drivers of species distributions, allowing for flexible covariate relationships that can incorporate modeling of optimal ranges and thresholds (Brodie et al., 2020). In addition, they can be used to account for spatial autocorrelation by modeling unexplained spatial variation in counts using a smoothing function. When fitting our full model, we incorporated five components: (1) estimates of relationships with spatial covariates, (2) a spatial random effect to estimate patterns and uncertainty related to unexplained spatial variation, (3) survey‐specific covariates to account for and understand temporal variation in observations, (4) a site‐level random‐effect to control for multiple visits to the same survey location, and (5) adjustments using offsets to account for survey‐specific measures of sampling area and effort that differed among sample records based on differences in survey design. To organize our data and assign replication of observations when more than one sample event occurred in the same area, we gridded our study area into 100 by 100 m (1 ha) grid cells. Covariates are generated for each cell and predictions are made for each individual cell.

We fit all GAM models in the mgcv package (Wood et al., 2016) in R (R Core Team, 2022). For three environmental covariates (elevation, HLI, IMI), we fit a separate smooth for each variable using a thin‐plate spine with *k* = 6 knots. We expected these covariates to affect both *P. shenandoah* and *P. cinereus* on the small scale of our overall sampling area. For our temporal environmental covariates, we fit a bivariate smoothing function for 3‐day precipitation by 10‐day average temperature. This allowed us to flexibly estimate interactions between the two variables. We predicted that counts would peak when conditions were both cool and wet, and this function allowed us to estimate the joint effect of the two environmental variables while also allowing for thresholds and optimized relationships to be estimated. For these variables, we fit Duchon splines and included *k* = 14 knots. Finally, we included a smooth effect of spatial location (e.g., across latitude and longitude, measured in UTMs) to account for spatial dependency among the expected counts, again fitting a Duchon spline to allow for finer‐scale spatial variation to be captured by the variable (settings for knots are addressed in the next section). In addition, we included a random effect of site to account for multiple visits if they occurred to the same site. All smooths and random effects were fit using quadratic penalties to regularize estimates and avoid overfitting using the default settings for the mgcv package. Finally, we included log(area) as an offset, to standardize observations so that expected counts are adjusted by the area surveyed under each protocol. We measured area in hectares, meaning estimates from the model are the number of salamanders expected to be counted per hectare of sampled area. Given imperfect detection, the counts are a subset of the total population. Given previous studies of marked populations of similar species, true densities are likely in the range of 1–2 orders of magnitude greater than the observed counts (Bailey et al., 2004a, 2004b; Sanchez et al., 2020).

Our overall model for the expected number of observed salamanders per hectare, C, at site *i* and time *t* was modeled as a zero‐inflated Poisson variable:

$$\log\left(C_{\mathrm{it}}\right)\sim \beta_{1}\left({\text{elev}}_{i}\right)+\beta_{2}\left({\mathrm{HLI}}_{i}\right)+\beta_{3}\left({\mathrm{IMI}}_{i}\right)+\beta_{4}\left({{\text{precip}}_{\mathrm{it}}}^{*}{\text{temp}}_{\mathrm{it}}\right)+\beta 5\left({{\text{latitude}}_{i}}^{*}{\text{longitude}}_{i}\right)+\log\left({\text{Area}}_{\mathrm{it}}\right)+\delta_{i},$$

where the *β*_1‐5_ are the estimated smoothed functions and *δ*_*i*_ is a random effect of site. The interaction for latitude and longitude allowed us to model spatial variation across both simultaneously. Including temperature and precipitation together, we allowed for non‐additive effects between the two variables, which we hypothesized would be the case if high surface activity required both to be maximized simultaneously.

### 2.3 Cross validation

To determine optimal settings for our spatial random effect, we fit the model with three different allowed maximum knots (25, 50, and 200) and compare their predictive power, coefficient, and effect size estimates. Our main goal was to make the most robust predicted range map. Therefore, we used blocked cross validation to compare models by removing 1/9th of the sampled area and then predicting to the removed area to assess the predictive ability of each model. Since the location of the removed area may matter, we repeated this for each of the 9 data subsets, re‐fit and re‐predicted, and took the cumulative best‐predicting model across all 9 subsets. To check predictions, we use root mean square error (RMSE) to compare among models following Althouse et al. (2011), and calculated coverage at the 95% CI.

### 2.4 Range size

We calculated range size based on two cut‐off values. The first is the more liberal calculation. For this we included any cell for which the *P. shenandoah* mean expected count is ≥1 salamander per hectare under average environmental conditions, regardless of uncertainty in the count. This estimate represents an upper extent of the range size, incorporating all of the areas estimated to potentially occur in the species' range. Alternatively, we calculated a conservative metric of minimum range size, where we only included locations where the lower confidence interval (i.e., estimated values two standard errors below the estimated mean) is greater than one. This represents an area where we estimated that with high certainty sites actually contain *P. shenandoah*. The first method was more likely to include sites near the range periphery where more uncertainty occurred, where the second method primarily included sites in the core of the range with little uncertainty about range boundaries.

### 2.5 Field sampling effects on map accuracy

In addition to generating updated estimates of occupied locations and range extent, we wished to determine how decisions regarding sampling design and effort affected our inference. We used a simulation study to accomplish this, using the data we collected and sub‐sampling to allow us to explore how sampling decisions influenced our results (DiRenzo et al., 2023). We focused on two outputs from our model: the estimate of overall range size, and our estimates of covariate relationships. For each scenario, we used cross validation as described above to find the best predictive model.

We investigated four different scenarios. Each of these four scenarios allowed us to examine the effect of reducing either spatial coverage or temporal replication, and to identify the optimal strategy for site selection across an environmental gradient. For the first scenario, we reduced the sampled area by two‐thirds, leaving one‐third of the unique sampled grid cells (*n* = 127) from the full data set (*n* = 383). We chose sampled sites that maximized sampling variation across the entire elevation gradient, an a priori hypothesis of a strong driver of *P. shenandoah* presence. For the second scenario, we again reduced our overall spatial sampling to one‐third of the total sampled sites, this time instead selecting sites based on maximizing the representation of three covariates: HLI, IMI, and elevation. This was calculated by maximizing the Euclidean distance in covariate space (maximal intra‐cluster space) among the sampled sites. To do this we calculated Euclidean distance on 10,000 random draws of sets of sites and took the set with the maximum out of 10,000. For the third scenario, instead of reducing number of sites, we reduced the number of sampling events to only include the first survey event per year which left 1347 observations, 11% of the original data (all sites included n = 383). The fourth scenario represents a spatial bias in site selection; we only included sampled sites within 250 m of a road or trail (*n* = 255).

## 3 RESULTS

The models for both *P. shenandoah* and *P. cinereus* had the greatest predictive accuracy when we used *k* = 200 knots in the spatial smooth (Table S1). Thus, the reported estimates below are from those two models. All other model estimates can be found in the supplement (Figures S1–S4).

### 3.1 Covariate estimates

**FIGURE 1:**
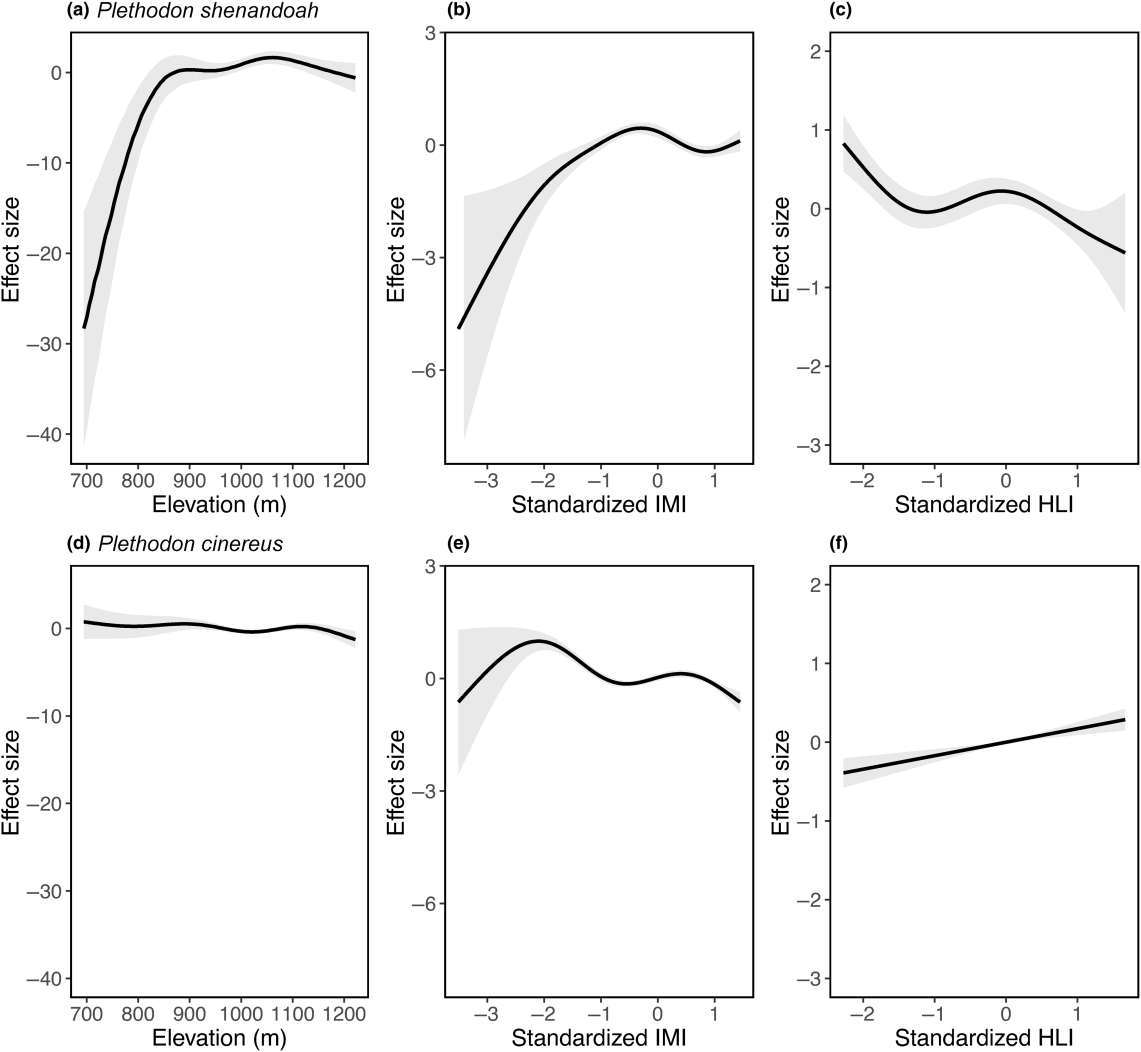
Effect size of elevation (Panels a, d), IMI (‘Integrated Moisture Index’ Panels b, e), and HLI (‘Heat Load Index’ panels c, f) on *Plethodon shenandoah* (panels a–c) and *Plethodon cinereus* (panels d–f) counts. Envelopes are standard error on the effect size. Please note that y‐axes are standardized within covariate but not across covariates (i.e, *y*‐axis for both elevations are the same but not between elevation and IMI.)

**FIGURE 2:**
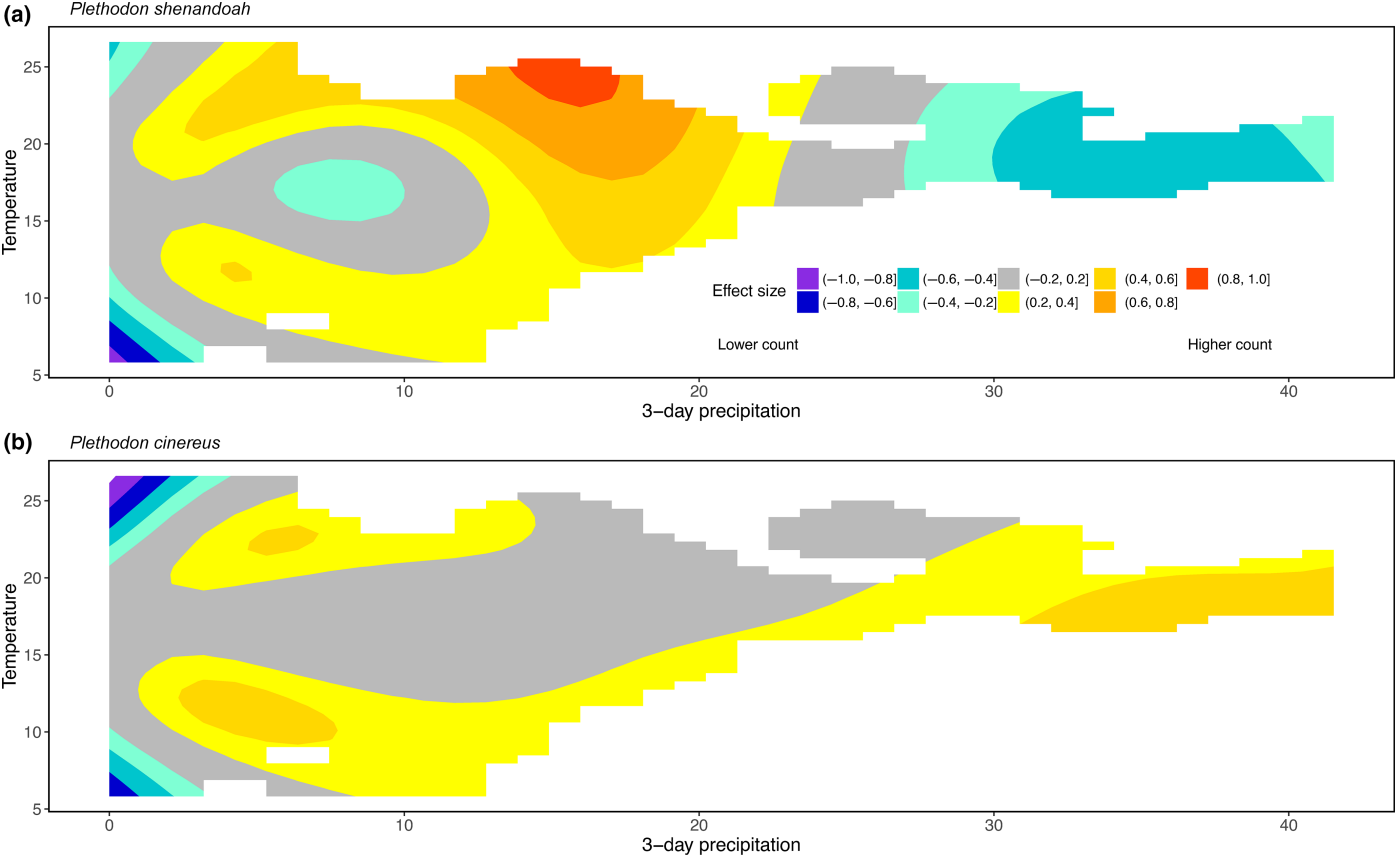
Effect size of temperature and precipitation on *Plethodon shenandoah* (panel a) and *Plethodon cinereus* (b).

For *P. shenandoah*, we found that expected counts quickly decline once sites drop below a threshold elevation of about 850 m (Figure 1a). *P. shenandoah* densities peak at locations with mean IMI (Figure 1b), and at lower values of HLI (Figure 1c). These all agreed with field observations (Figure S10). We found that surveys that occur on days with low to intermediate precipitation (between 5 and 20 mm over the previous 3 days) were predicted to have higher *P. shenandoah* detections than when surveys occurred after periods of low or high precipitation (Figure 2a). We found the highest effect size for temperature between 17 and 25°C (Figure 2a). Deviance explained for this model is 57.1%.

For *P. cinereus*, we found very small negative effects of elevation on expected counts (Figure 1d). Compared to *P. shenandoah, P. cinereus* had higher counts at lower IMI, with slight declines as IMI increased (Figure 1e). Unlike *P. shenandoah*, high values of HLI were associated with greater *P. cinereus* counts (Figure 1f). *P. cinereus* had fairly consistent detectability across temperature when there had been at least some precipitation in the previous 3 days (Figure 2b). Deviance explained for this model is 53.9%.

### 3.2 Mapped ranges

**FIGURE 3:**
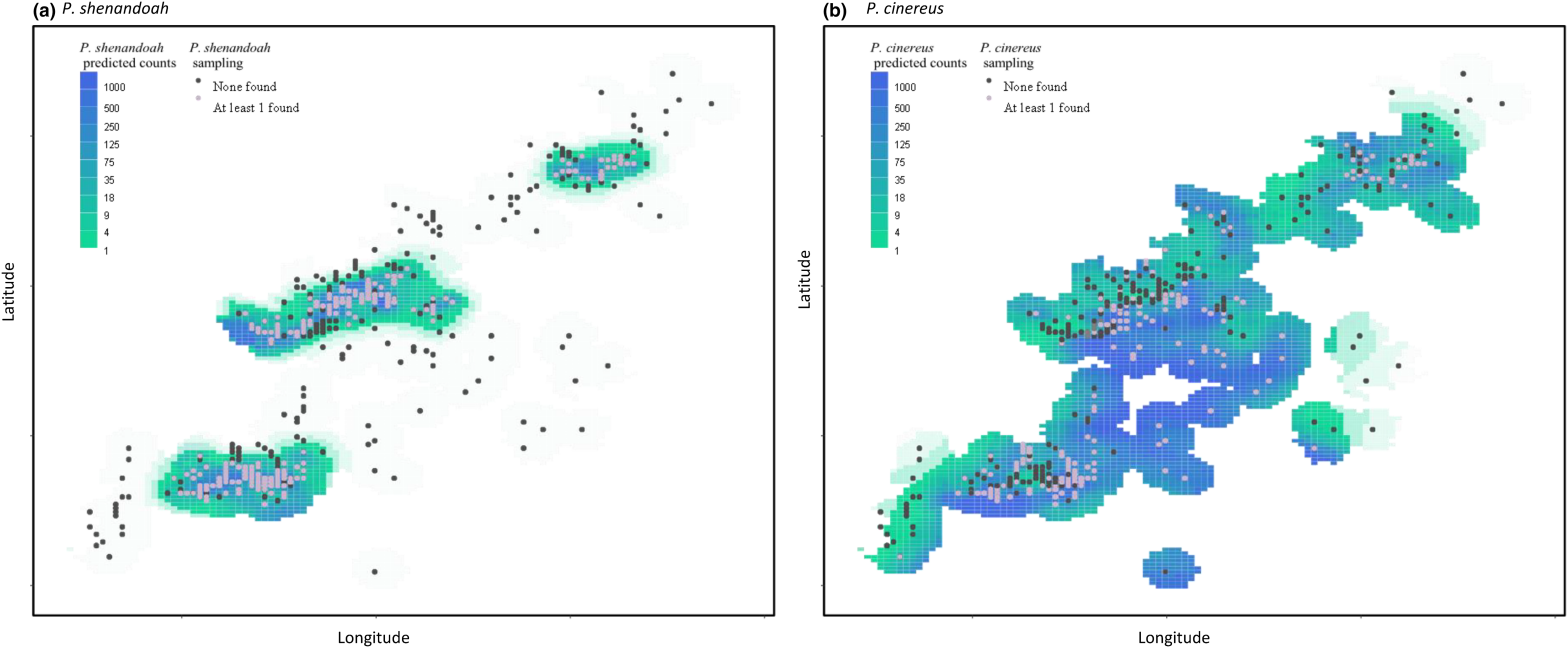
Map of *Plethodon shenandoah* (Panel a) and of *Plethodon cinereus* predicted counts (b).

For *P. shenandoah*, our best model estimated an area of 8.67 km^2^ with a mean expected count of at least one individual (Figure 3a; Table S2). Predicted counts are standardized for an area of 1 ha and average conditions. Thus, this threshold included all locations that would be predicted to have at least one observed salamander if all cover objects in the one‐hectare area were searched. This represents a maximum range extent for the species. Alternatively, if we used a threshold of only including areas where the lower confidence interval for expected counts is greater than one, the models estimated only 6.02 km^2^ as the maximum range extent. We found that *P. cinereus* is expected to have mean expected counts greater than one in 34.6 km^2^, which is 79.5% of the surveyed region (Figure 3b; Table S2 – note the full range of this species extends throughout much of the forested northeast of North America, and this estimate only includes the part of the species range within our study area). There were <9 km^2^ where we expected counts of *P. cinereus* to be less than one individual. Overall, as expected, error in estimated counts was greatest in areas where *P. shenandoah* has never been observed, and where previous sample effort has been minimal (Figure 3a.).

**FIGURE 4:**
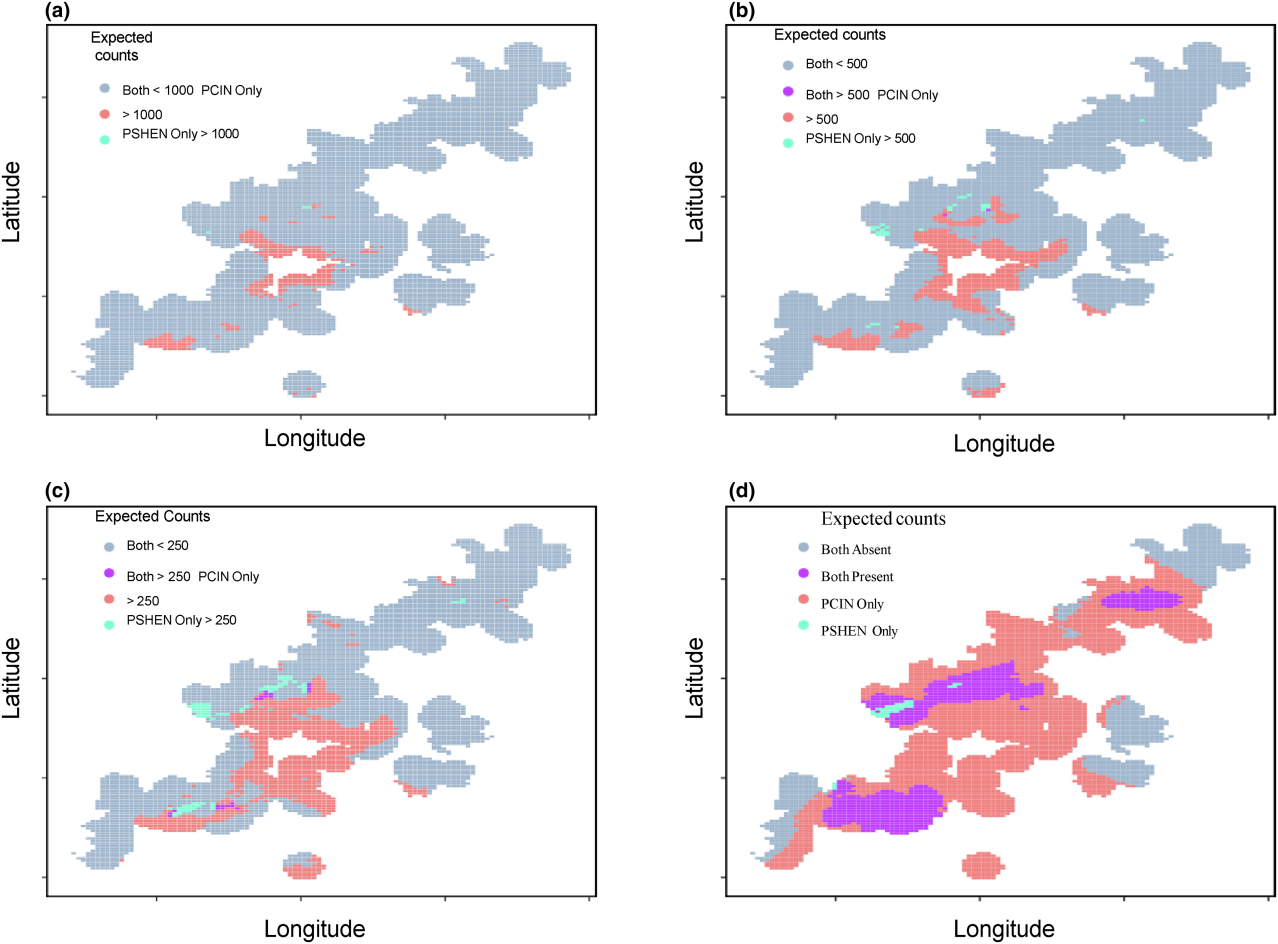
Areas where *Plethodon shenandoah* (PSHEN) and *Plethodon cinereus* (PCIN) mean counts are expected to be over 1000 (panel a), 500 (b), 250 (c), or at least 1 (d) individuals.

We found that in most of *P. shenandoah'*s range (i.e., within the 8.67 km^2^ where the mean expected count was greater than one) we expect P. *cinereus* to also be present (Figure 4d). We only found 0.37 km^2^ where we expect *P. shenandoah* to be present alone. In cells where counts of *P. cinereus* are predicted to be high (>1000 individuals) or where *P. shenandoah* is expected to be high (>1000), we never predict the other species to also have a high count (Figure 4a), suggesting that while overlap occurs, that the core of each range does not overlap. As expected, as counts decrease (<500, <250), we expect more and more area where both species have higher counts (Figure 4b,c).

### 3.3 Field sampling effects on map accuracy

Model fits for sub‐set data models were relatively comparable to using the full dataset (Table S3).

Estimates of the effect of elevation on expected counts of *P. shenandoah* were fairly consistent across our four scenarios (Figure S5), with exception of when the subset was selected based on proximity to a road or trail, where the model estimated little effect of elevation (Figure S5E). This corresponds to low variation in sampled elevation, as most trails and roads are constrained to a limited elevational breadth.

Estimates of the effect of IMI on expected counts on *P. shenandoah* were consistent across subsets. Across subsets, estimates for the effect of HLI on expected counts on *P. shenandoah* were reasonably consistent (Figure S7) except for when data were subset by maximizing across three covariates (Panel C), where the model estimated strong negative effects of high HLI.

All four simulated sampling scenarios had similar estimates of the effect of precipitation and temperature on expected counts of *P. shenandoah*, with small changes in effect size across precipitation and temperature (Figure S8). The only major deviation is when only a single visit per year was used. In this scenario low precipitation was estimated to be positively correlated with salamander counts.

With three of four reduced data sets, we estimated a slightly larger occupied range for *P. shenandoah* than what we predicted when the full data set was used (Table S4). However, the estimates of where we expect *P. shenandoah* to occur were quite consistent across subsets even if specific count estimates were variable (Figure S9). We also had higher uncertainty in counts when the available survey data were reduced (Figure S9), except under a scenario where each site was visited only once annually. In this scenario, the model results were overconfident in the estimated counts (Panel E).

## 4 DISCUSSION

Uncertainty in range maps comes from a variety of sources, including model decisions and field sampling protocol. Here we explored how various forms of uncertainty affect our understanding of the driving forces of *P. shenandoah*'s range, and the estimated range size and location. Our results support previous work that *P. shenandoah* is likely restricted to a small portion of the available high‐elevation habitat within Shenandoah National Park. Across all model choices and all field sampling scenarios, the greatest predicted occupied area was 17.3 km^2^ (Tables S2 and S4), and the best estimates of the range size are between 6.02 and 8.67 km^2^, suggesting a robustness to the inference that the range of *P. shenandoah* is small. Previous estimates for the range size were under 4 km^2^ but did not report error and were based on much more limited surveys and assumptions about presumed occurrence where sampling did not occur. This is the first comprehensive re‐evaluation of the range since the original maps (USFWS, 1994).

The National Park Service has a mandate to both conserve habitats within park boundaries, and to protect Endangered Species. Occasionally these mandates are at odds. Our results provide a quantification of the uncertainty in the range of an Endangered Species such that the National Park Service can make informed management decisions for both *P. shenandoah* and other park resources.

The range of the congeneric *P. cinereus* is estimated to overlap much of *P. shenandoah's* range (Figure 4). Whether this has been true historically is hard to know, as detailed maps of *P. cinereus* in the region are unavailable. Previous work inferred that *P. shenandoah* habitats were occupied by the species alone, though they were expected to be invasible by the presumably dominant competitor *P. cinereus* (Jaeger, 1971). Recent genetic work between *P. cinereus* and a different mountain endemic, *P. hubrichti*, suggests that movement of *P. cinereus* into its range is relatively recent (Page et al., 2020), so it is possible that is the case for the *P. shenandoah* occupied range as well.

Differences in the effect of covariates on *P. cinereus* and *P. shenandoah* may suggest niche partitioning as seen in other places where *P. cinereus* occurs with other plethodontid salamander species (e.g., Farallo & Miles, 2016), and opens the question as to how much the two species are currently competing given both different (and sometimes opposite) drivers of occupancy and abundance. Indeed, recent work has struggled to find evidence to support a hypothesis of interspecific competition (Amburgey et al., 2019; Grant et al., 2018). We found that *P. cinereus* and *P. shenandoah* have different relationships with elevation, such that *P. shenandoah* is more likely to occur at high elevations while *P. cinereus* is more likely at the lower elevations in the study area; this was also observed by Grant et al. (2018), who provided evidence that the elevation limit may be controlled by the cloud base height. Cloud base height may provide transient moisture in otherwise inhospitable habitats in which *P. cinereus* has more difficulty persisting, evidence of potential niche (i.e., environmental moisture) partitioning. Despite this potential partitioning, we estimated that *P. cinereus* occurs at much higher densities across the high‐elevation habitat than *P. shenandoah*.

While it might be promising in light of climate change that *P. shenandoah* doesn't seem limited by current temperatures, (i.e., 16.8°C ± 3.9 standard deviation; maximum 10 day average 26.6°C and minimum 10 day average 5.6°C), it is unknown whether *P. shenandoah* physiological limits will be exceeded in climate change scenarios, expected to increase by 2 to 4.2°C per decade (Lee et al., 2014), or if they are able to adapt to changing temperatures. *P. cinereus* is expected to be strongly negatively affected by temperature increases, as their use of surface resources decreases at high temperatures (Sanchez et al., 2020). In fact, the differential competitive advantage of *P. cinereus* is expected to shrink with increased temperatures (Dallalio et al., 2017). However, *P. cinereus* may also have high capacity for genetic adaptation (Adams et al., 2007), though its ability to adapt to rapidly changing temperature or drying conditions, expected in a future climate for the high‐elevation habitat in Shenandoah National Park (Lee et al., 2014), is also unknown.

Overall, estimates of the effects of covariates were somewhat dependent on model choice and field sampling method. We believe this to be a critical result—if we would have had a less‐complete data set, without the diversity of sampling locations and methods, we would have misunderstood the interaction between the covariates and *P. shenandoah* counts, leading to biased estimates of the species range. When sites were selected along a single a priori covariate (e.g., elevation), we got different estimates for the effect of IMI. Indeed, even when sites were selected across three a priori covariates, the effect of IMI (and to a lesser extent HLI) was estimated to be quite different than when sampling occurred over the entire range of the salamander. In a more extensive discussion of study design for mapping soil types, Ma et al. (2020) likewise show that how sites are selected across a physical gradient changes resulting inference. They also find that including critical features is important to create accurate maps; in particular they suggest that sites should be selected by minimizing distances to important features (‘feature space coverage’) rather than by random selection or by selecting across a correlation matrix of features (e.g., Latin hypercube; Ma et al., 2020). For *P. shenandoah*, the ability to survey across the entire range allowed for comparisons between partial sampling of a range and full range sampling. We can extend this inference to other species with larger geographic ranges; our example suggests that it is preferable to have more sites over the range than to have repeat visits for accurate covariate estimates and range size estimates.

## AUTHOR CONTRIBUTIONS

**Jo A. Werba:** Data curation (supporting); formal analysis (lead); writing – original draft (lead); writing – review and editing (lead). **Evan H. Campbell Grant:** Conceptualization (lead); funding acquisition (lead); project administration (lead); writing – original draft (supporting); writing – review and editing (supporting). **Adrianne B. Brand:** Data curation (lead); methodology (supporting); project administration (supporting); writing – original draft (supporting); writing – review and editing (supporting). **David A. W. Miller:** Formal analysis (equal); methodology (lead); writing – original draft (supporting); writing – review and editing (supporting).

## Supporting information


Data S1

## Data Availability

All code and data are publicly available at NEARMI/Range_Map_Public: Code for creating P. shenandoah range map (github.com). We will permanently store code at Zenodo upon acceptance. Data provided with code do not contain the sensitive geographical information on an endangered species. Field and covariate data are separately published in Grant et al. (2024) with obscured spatial location information.

## Appendix

**TABLE A1:** Individual source details for occurrence data on *Plethodon shenandoah* and *Plethodon cinereus* from 2007 to 2022 included in our study.

Data source/study	Years active	Sampling site design	Survey type	Number of sites sampled
Sevin (2014)	2007	4 × 4 m plot, 8 × 8 m plot, 32 × 2 m transect	Daytime active cover, nighttime visual encounter	16
Sevin (2014)	2008, 2009	32 × 2 m transect	Daytime active cover, nighttime visual encounter	124
Grant et al. (2024)	2009	Series of 4–8 4 × 4 m plots, spaced 10 m apart	Daytime active cover	6
High‐elevation Occupancy	2011–2014, 2016	2 perpendicular 32 × 2 m transects	Daytime active cover	131
Grant et al. (2024)	2012	Series of 4–8 parallel 32 × 2 m transects, spaced 10 m apart	Daytime active cover	6
Grant et al. (2018)	2011, 2013	2 perpendicular 50 × 2 m transects	Daytime active cover	51
Amburgey et al. (2019, 2020)	2015, 2016	50 × 2m transects	Daytime active cover	62
Dallalio et al. (2017)	2012	100 × 10 m transects	Daytime active cover	11
Grant et al. (2024)	2015	50 × 2 m transects downslope	Nighttime visual encounter	30
Grant et al. (2024)	2013, 2014	2 side‐by‐side parallel 32 × 2 m transects	Daytime active cover	31
Grant et al. (2021)	2019–2022	2 perpendicular 32 × 2 m transects	Daytime active cover	150

**TABLE A2:** Overlap in sampled locations among studies 2007–2022 on occurrence of *Plethodon shenandoah* and *Plethodon cinereus*.

Number of overlapping study sites	Sevin (2014)	Sevin (2014)	Grant et al. (2024)	High‐elevation occupancy	Grant et al. (2024)	Grant et al. (2018)	Amburgey et al. 2019, (2020)	Dallalio et al. (2017)	Grant et al. (2024)	Grant et al. (2024)	Grant et al. (2021)
Sevin (2014)	**16**	—	—	—	—	—	—	—	—	—	—
Sevin (2014)	16	**124**	—	—	—	—	—	—	—	—	—
Grant et al. (2024)	1	1	**6**	—	—	—	—	—	—	—	—
High‐elevation occupancy	5	46	0	**131**	—	—	—	—	—	—	—
Grant et al. (2024)	0	0	6	0	**6**	—	—	—	—	—	—
Grant et al. (2018)	1	1	0	1	0	**51**	—	—	—	—	—
Amburgey et al. 2019, (2020)	3	6	4	2	4	0	**62**	—	—	—	—
Dallalio et al. (2017)	2	2	0	0	0	0	5	**11**	—	—	—
Grant et al. (2024)	0	1	0	0	0	0	0	0	**30**	—	—
Grant et al. (2024)	0	1	0	0	0	0	0	0	3	**31**	—
Grant et al. (2021)	3	21	0	19	0	4	4	0	0	0	**150**

*Note*: The values in boldface (on the diagonal) are the number of sample locations for a given dataset.
