# Supplementary material for: Updated range map of an endangered salamander and congeneric competitor reveals different niche preferences
Source: Ecol Evol. 2024 May 20;14(5):e11262. doi: 10.1002/ece3.11262 (PMC11106042; doi:10.1002/ece3.11262)
Supplement: Supplementary file 1 — Data S1 [file ECE3-14-e11262-s001.docx]

Supplement: Updated range map of an endangered salamander and congeneric competitor reveals different niche preferences

#### Jo A. Werba^a,b^, David A.W. Miller^b^, Adrianne B. Brand^a^, Evan H. Campbell Grant^a^

#### ^a^ *U.S. Geological Survey, Eastern Ecological Science Center (Patuxent Wildlife Research Center), SO Conte Anadromous Fish Research Laboratory, 1 Migratory Way, Turners Falls, MA 01376, USA.*

*^b^ Department of Ecosystem Science and Management, Penn State University, University Park, PA 16802*

**Contents**

List of Figures 2

1. Covariate Figures 3
2. Site subsets 7
3. Raw Data Figures 12
4. Covariate Correlation 14
5. Supplemental Tables 15

# List of Figures

S1 Effect size of elevation (A., D.), Integrated Moisture Index (IMI; B., E.) and Heat Load Index (HLI; C., F.) on *Plethodon shendandoah* (A., B., C.) and *P. cinereus* (D., E., F.) counts. For models with 25 spatial knots. Envelopes are standard error on the effect size. Please note that y-axis is standardized within covariate but not across covariates (i.e, y-axis for both elevations are the same but not between elevation and

IMI.) 3

S2 Effect size of temperature and precipitation on *Plethodon shenandoah* (A.) and *P. cinereus* (B.). For models with 25 spatial knots 4

S3 Effect size of elevation (A., D.), Integrated Moisture Index (IMI; B., E.) and Heat Load Index (HLI; C., F.) on *Plethodon shendandoah* (A., B., C.) and *P. cinereus* (D., E., F.) counts. For models with 50 spatial knots. Envelopes are standard error on the effect size. Please note that y-axis is standardized within covariate but not across covariates (i.e, y-axis for both elevations are the same but not between elevation and

IMI.) 5

S4 Effect size of temperature and precipitation on *Plethodon shenandoah* (A.) and *P. cinereus* (B.). For models with 50 (A.) and 200 (B.) spatial knots 6

S5 Elevation effect size estimates for *Plethodon shenandoah* given full dataset (A.), subset dataset by elevation (B.), eleva- tion, Integrated Moisture Index (IMI) and Heat Load Index (HLI; C.), by reducing dataset to only a single visit per year (D.) or by distance to roads and trails (E.). Please note that due to highly variable error the y-axes are not consistent

across panels. 7

S6 Integrated Moisture Index (IMI) effect size estimates for *Plethodon shenandoah* given full dataset (A.), subset dataset by elevation (B.), elevation, IMI and Heat Load Index (HLI; C.), by reducing dataset to only a single visit per year (D.) or by distance to roads and trails (E.). Please note that due to high estimates the y-axis for panel C is different from the

other panels. 8

S7 Heat Load Index effect size estimates for *Plethodon shenandoah* given full dataset (A.), subset dataset by elevation (B.), elevation, Integrated Moisture Index (IMI) and HLI (C.), by reducing

dataset to only a single visit per year (D.) or by distance to roads and trails (E.).

9

S8 Effect size of precipitation and temperature on *Plethodon shendandoah* counts given full dataset (A.), subset dataset by elevation (B.), elevation, Integrated Moisture Index (IMI) and Heat Load Index (HLI; C.), by reducing dataset to only a single visit per year (D.)

or by distance to roads and trails (E.). 10

S9 *Plethodon shenandoah* range estimates given full dataset (A.), subset dataset by elevation (B.), elevation, Integrated Moisture Index (IMI)and Heat Load Index (HLI; C.), by reducing dataset to only a single visit per year (D.) or by distance to a road or trail (E.). Colors represent mean estimates and opacity shows confidence in the estimate.

The more opaque the color the more confident in the mean estimate. 11

S10 Raw *Plethodon shenandoah* count data against covariates used in models, including Heat Load Index (HLI), elevation, and Integrated Moisture Index (IMI) 12

S11 Raw *Plethodon cinereus* count data against covariates used in models, including Heat Load Index (HLI), elevation, and Integrated Moisture Index (IMI) 13

S12 Covariate correlations, including standardized Heat Load Index (HLI), elevation (elev), and Integrated Moisture Index (IMI) 14

# Covariate Figures

This section contains covariate estimate figures from models fit with a sub-optimal number of knots in the spatial smooth.

Figure S1: Effect size of elevation (A., D.), Integrated Moisture Index (IMI; B., E.) and Heat Load Index (HLI; C., F.) on *Plethodon shendandoah* (A., B., C.) and *P. cinereus* (D., E., F.) counts. For models with 25 spatial knots. Envelopes are standard error on the effect size. Please note that y-axes are standardized within covariate but not across covariates (i.e,, y-axis for both elevations is the same but not between elevation and IMI.)

- 1. *P. shenandoah* B. C.

5

0

0 0

−5

−10 −5

Effect Size

Effect Size

Effect Size

−2

−15

−20

0

700 800 900 1000 1100 1200

Elevation (m)

D. *P. cinereus*

−10

−15

5

−3 −2 −1 0 1

Standardized IMI

E.

−4

−2 −1 0 1

Standardized HLI

F.

0 0

−5

−10 −5

Effect Size

Effect Size

Effect Size

−2

−15

−20

700 800 900 1000 1100 1200

Elevation (m)

−10

−15

−3 −2 −1 0 1

Standardized IMI

−4

−3 −2 −1 0 1

Standardized HLI

Figure S2: Effect size of temperature and precipitation on *Plethodon shenandoah* (A.) and *P. cinereus* (B.). For models with 25 spatial knots.

A*. P. shenandoah*

25


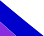

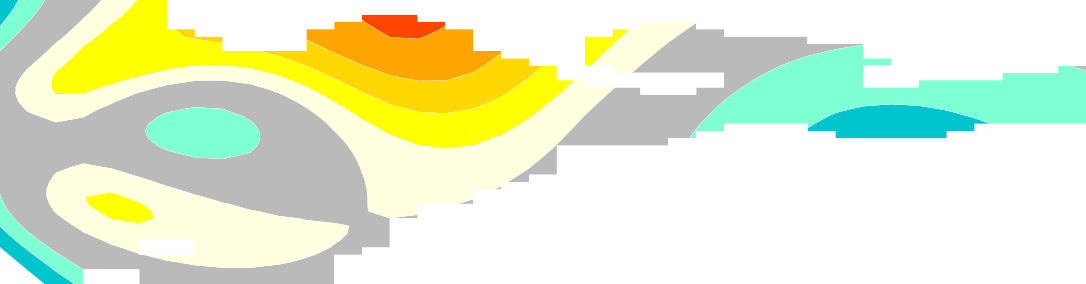

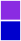

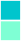

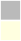

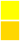

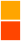


(−1.0, −0.8] (−0.6, −0.4] (−0.2, 0.2] (0.4, 0.6] (0.8, 1.0]

Effect Size

(−0.8, −0.6] (−0.4, −0.2] (0.2, 0.4] (0.6, 0.8] (1.0, 1.2]

Lower count

Higher count

Temperature

20

15

10

5

0 10 20 30 40

3−day precipitation

B. *P. cinereus*

25


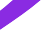


Temperature

20

15

10

5

0 10 20 30 40

3−day precipitation

Figure S3: Effect size of elevation (A., D.), Integrated Moisture Index (IMI; B., E.) and Heat Load Index (HLI; C., F.) on *Plethodon shendandoah* (A., B., C.) and *P. cinereus* (D., E., F.) counts. For models with 50 spatial knots. Envelopes are standard error on the effect size. Please note that y-axis are standardized within covariate but not across covariates (i.e, y-axis for both elevations are the same but not between elevation and IMI.)

A*. P. Shenandoah* B. C.

1

1. 0

−5 −1 0

−10 −2

Effect Size

Effect Size

Effect Size

−1

−15 −3

−2

−20 −4

700 800 900 1000 1100 1200

Elevation (m)

D. *P. cinereus*

0

−3 −2 −1 0 1

Standardized IMI

E.

0

−2 −1 0 1

Standardized HLI

F.

1

−5 0

−1

Effect Size

Effect Size

Effect Size

−10

−2 −1

−15

−20

700 800 900 1000 1100 1200

Elevation (m)

−3

−4

−3 −2 −1 0 1

Standardized IMI

−2

−2 −1 0 1

Standardized HLI

Figure S4: Effect size of temperature and precipitation on *Plethodon shenandoah* (A.) and *P. cinereus* (B.). For models with 50 (A.) and 200 (B.) spatial knots

A. *P. shenandoah*

25


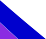

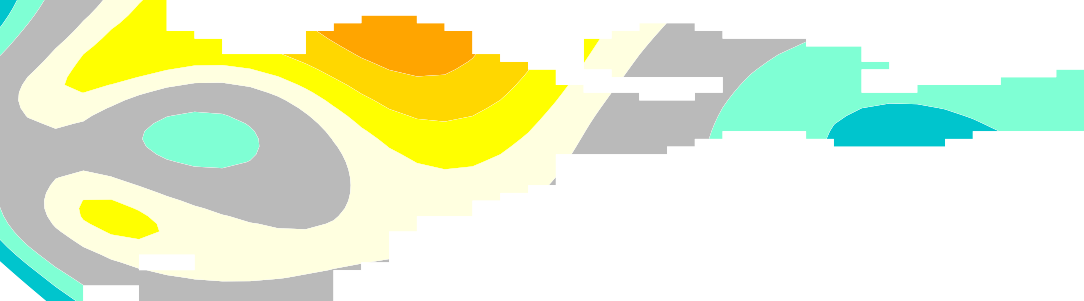

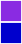

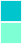

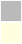

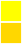


(−1.0, −0.8] (−0.6, −0.4] (−0.2, 0.2]

(−0.8, −0.6] (−0.4, −0.2] (0.2, 0.4]

(0.4, 0.6]

(0.6, 0.8]

(0.8, 1.0]

Effect Size

Lower count

Higher count

20

Temperature

15

10

5

0 10 20 30 40

3 – Day Precipitation

B. *P. cinereus*

25

Temperature

20

15

10

5

0 10 20 30 40

3 – Day Precipitation

# Site subsets

This section shows results from sub-setting the complete dataset into different possible ways a land area may be likely to be surveyed for *Plethodon shenandoah*.

Figure S5: Elevation effect size estimates given full dataset (A.), subset dataset by elevation (B.), elevation,
Integrated Moisture Index (IMI) and Heat Load Index (HLI; C.), by reducing dataset to only a single visit per year (D.) or by distance to roads and trails (E.).
Please note that due to highly variable error the y-axis is not consistent across panels.

Figure S6: Integrated Moisture Index effect size estimates for *Plethodon shenandoah* given full dataset (A.), subset dataset by elevation (B.), elevation, Integrated Moisture Index (IMI) and Heat Load Index (HLI; C.), by reducing dataset to only a single visit per year (D.) or by distance to roads and trails (E.). Please note that due to high estimates the y-axis for panel C is different from the other panels.

- - 1. Full dataset
    2. Subset by elevation
    3. Subset by elevation, HLI an

0

0 0

−10

−20

Effect Size

−20

−20

−40

−40

−30

−3 −2 −1 0 1

- - 1. Single visit by year

−2 −1 0 1

- - 1. Subset by Distance

−40

−2 −1 0 1

Standardized IMI

0 0

−20 −20

Effect Size

−40 −40

−3 −2 −1 0 1

Standardized IMI

−3 −2 −1 0 1

Standardized IMI

Figure S7: Heat Load Index (HLI) effect size estimates for *Plethodon shenandoah* given full dataset (A.), subset dataset by elevation (B.), elevation, Integrated Moisture Index (IMI) and HLI (C.), by reducing dataset to only a single visit per year (D.) or by distance to roads and trails (E.).

1. Full dataset
2. Subset by elevation
3. Subset by elevation, IMII and HLI

5 5 5

0 0 0

−5 −5 −5

Effect Size

−10

−10

−10

−15

−2 −1 0 1

1. Single visit by year

−15

−2 −1 0 1

1. Subset by Distance

−15

−2 −1 0 1

Standardized HLI

5 5

0 0

−5 −5

Effect Size

−10 −10

−15

−2 −1 0 1

Standardized HLI

−15

−2 −1 0 1

Standardized HLI

Figure S8: Effect size of precipitation and temperature on *Plethodon shendandoah* counts given full dataset (A.), subset dataset by elevation (B.), elevation, Integrated Moisture Index (IMI) and Heat Load Index (HLI; C.), by reducing dataset to only a single visit per year (D.) or by distance to roads and trails (E.).


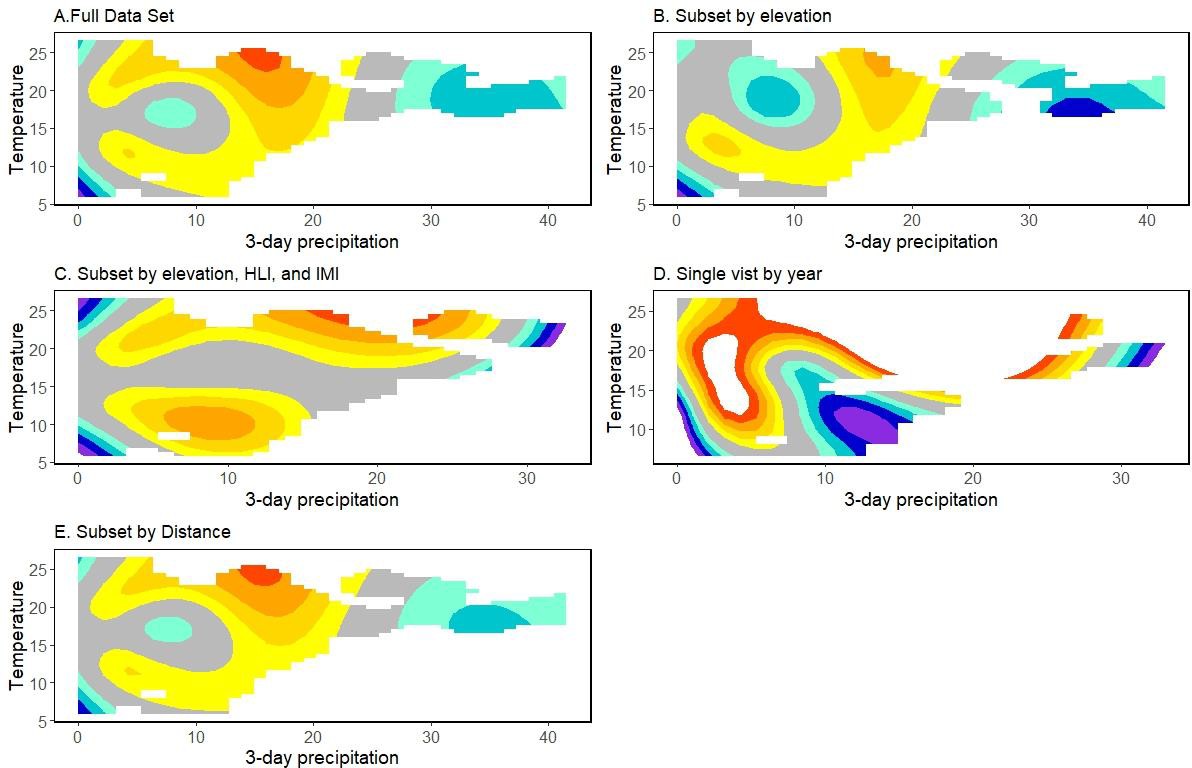

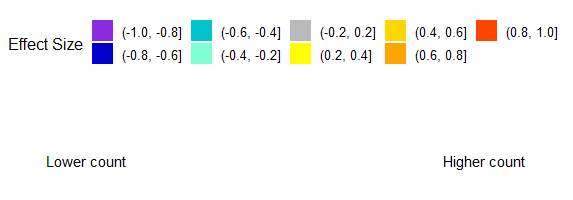


Figure S9: *Plethodon shenandoah* range estimates given full dataset (A.), subset dataset by elevation (B.), elevation, Integrated Moisture Index (IMI) and Heat Load Index (HLI; C.), by reducing dataset to only a single visit per year (D.) or by distance to a road or trail (E.). Colors represent mean estimates and opacity shows confidence in the estimate. The more opaque the color the more confident in the mean estimate.


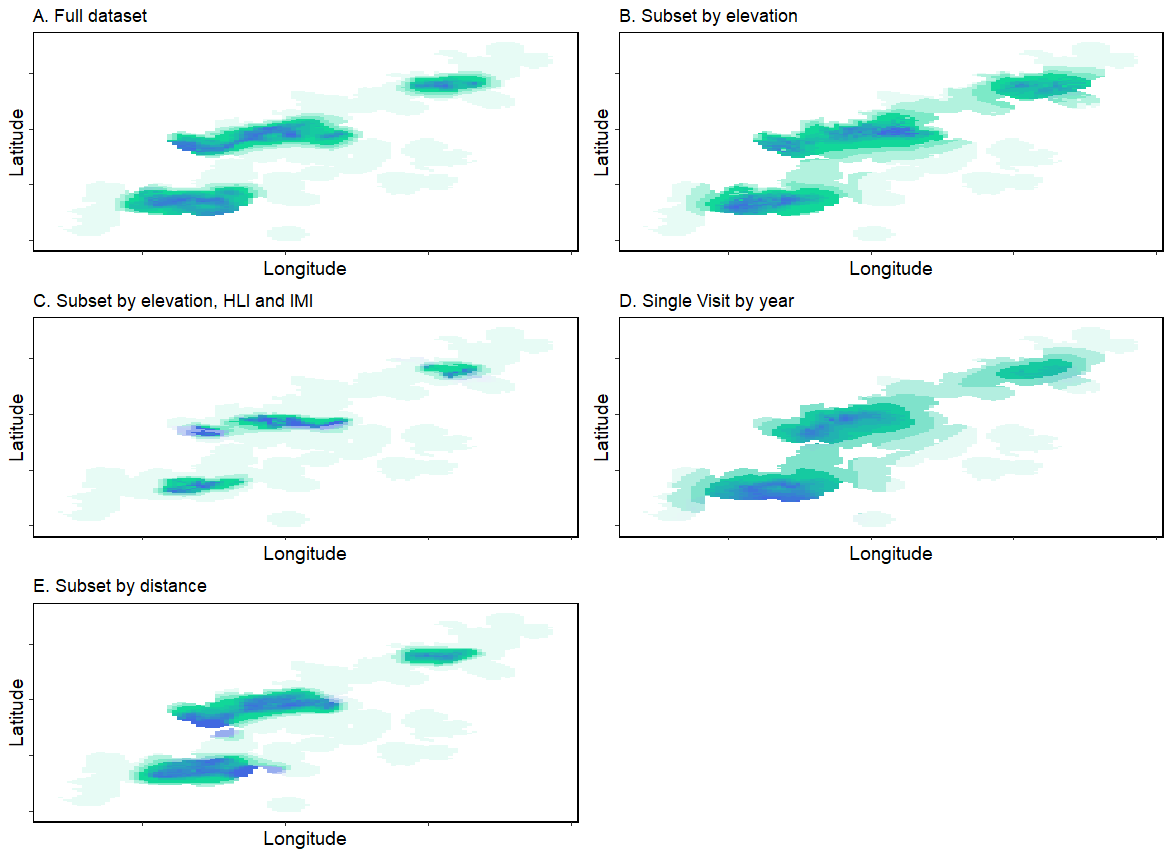

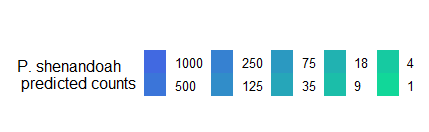


# Raw Data Figures

This section shows raw data from all surveys for the *Plethodon shenandoah* salamander (**Grant**, E.H.C., Miller, D.A.W, and Werba, J. 2024. An updated range map for Plethodon shenandoah: U.S. Geological Survey data release, https://doi.org/10.5066/P13OVUIC.).

Figure S10: Raw *Plethodon shenandoah* count data against covariates used in models, including elevation, Heat Load Index (HLI), and Integrated Moisture Index (IMI).

Figure S11: Raw *Plethodon cinereus* count data against covariates used in models, including elevation, Heat Load Index (HLI), and Integrated Moisture Index (IMI).

A.

*P. cinereus* count


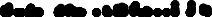

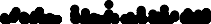

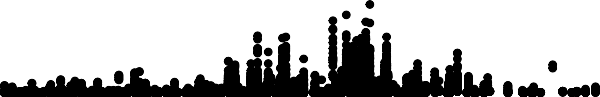


200

150

100

50

0

B.

*P. cinereus* count


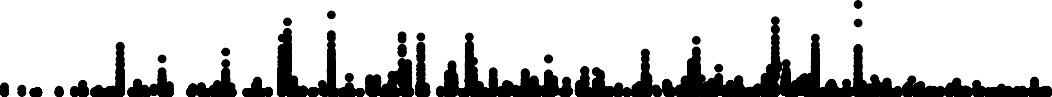


200

−1 0 1 2

Standardized Elevation

150

100

50

0

C.


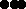

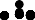

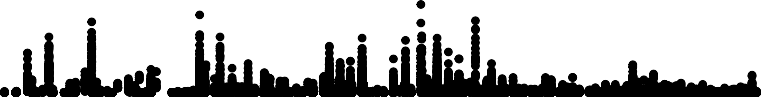


200

*P. cinereus* count

−2 −1 0 1

Standardized HLI

150

100

50

0

−3 −2 −1 0 1

Standardized IMI

# Covariate Correlation


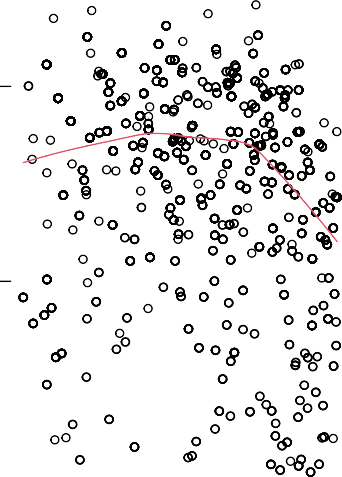


0.064

elev

−1.5

−1.0

−0.5

0.0

0.5

1.0

1.5

2.0

40

50

−2

−1

Figure S12: Covariate correlations, including standardized Heat Load Index (std_HLI), elevation (elev), and Integrated Moisture Index (IMI).

−1.5 −0.5 0.5 1.0 1.5 2.0

0.19

0.15

std_HLI

0

1

−2 −1 0 1 20 30 40 50


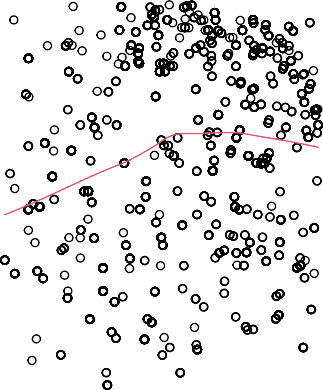

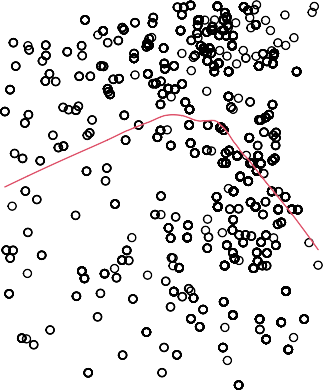


14

IMI

20

30

# Supplemental Tables

Here we present all tables to support an updated range estimate for *Plethodon shenandoah*.

| Species | Knots allowed | RMSE | RMSE with  single highest removed | Coverage | Coverage without  highest RMSE (percent) | Deviance  Explained (percent) |
| --- | --- | --- | --- | --- | --- | --- |
| *P. shenandoah* | 25 | 1*.*66*e*^31^ | 80.3 | 2.19 | 2.14 | 45.8 |
|  | 50 | 1*.*29*e*^28^ | 1523806 | 6.01 | 5.66 | 51.3 |
|  | 200 | 3*.*38*e*^8^ | 1059 | 23.3 | 19.6 | 57.1 |
| *P.cinereus* | 25 | 237 | 161 | 16.6 | 19 | 40.6 |
|  | 50 | 2155 | 155 | 45.3 | 49.5 | 45.4 |
|  | 200 | 2515 | 78.4 | 62.2 | 59.7 | 53.9 |

Table S1: Model fitness test results for different maximum knots allowed to estimate range of *Plethodon shenandoah* and *P. cinereus*. We calculated Root Mean Square Error (RMSE) across each cross-validation set and again with the highest RMSE removed since often a single fit drastically changed the mean RMSE.

| Species | Knots  allowed | Estimate (sq km) where  mean count is greater than 1 | Estimate (sq km) where  lower CI is greater than 1 |
| --- | --- | --- | --- |
| *P. shenandoah* | 25 | 17.31 | 13.4 |
|  | 50 | 10.24 | 8.2 |
|  | 200 | 8.67 | 6.02 |
| *P. cinereus* | 25 | 41.45 | 39.06 |
|  | 50 | 34.94 | 32.84 |
|  | 200 | 34.6 | 29.4 |

Table S2: Estimated range for different maximum knots allowed to estimate range of *Plethodon shenandoah* and *P. cinereus*.

| Sampling  Basis | Knots  allowed | | RMSE | | Coverage  (percent) | Deviance  Explained (percent) |
| --- | --- | --- | --- | --- | --- | --- |
| Elevation | 15 | | 865 | | 15.2 | 52.2 |
|  | 35 | | 58196215 | | 12.9 | 58.3 |
|  | 75 | | 352284 |  | 33.4 | 59.8 |
| Elevation-HLI-IMI | 15 | | 6*.*15*e*^11^ | | 6.79 | 66.3 |
|  | 35 | | 9*.*75*e*^9^ | | 21 | 70.8 |
|  | 75 | | 2*.*43*e*^15^ | | 27.2 | 74.2 |
| Single annual visit | 25 | | 28.6 | | 5.04 | 83.1 |
|  | 50 | | 24.7 | | 5.4 | 84.7 |
|  | 200 | | 40.8 | | 5.67 | 87.2 |
| Distance from roads and trails | 15 | | 7*.*41*e*^24^ | | 4.36 | 41.1 |
|  | 35 |  | 1*.*59*e*^95^ | | 2.2 | 49.1 |
|  | 75 |  | 9*.*09*e*^16^ | | 15.5 | 53.5 |

Table S3: Model fitness test results for different maximum knots allowed and subsets of data used to estimate range of *Plethodon shenandoah* and *P. cinereus*. We calculated Root Mean Square Error (RMSE) across each cross-validation set and again with the highest RMSE removed since often a single fit drastically changed the mean RMSE. Data were subset by elevation, elevation, Integrated Moisture Index (IMI) and Heat Load Index (HLI), by reducing dataset to only a single annual visit or by distance to roads and trails.

| Sub-set basis | Knots  allowed | Estimate (sq km) where  mean count is greater than 1 | Estimate (sq km) where  Lower CI is greater than 1 |
| --- | --- | --- | --- |
| Elevation | 75 | 9.1 | 5.67 |
| Elevation-HLI-IMI | 75 | 5.46 | 1.72 |
| Single annual visit | 200 | 9.56 | 4.1 |
| Distance | 75 | 9.26 | 6.7 |

Table S4: Estimated range area for subsets of data used to estimate range of *Plethodon shenandoah* and *P. cinereus*. Data were subset by elevation, elevation, Integrated Moisture Index (IMI) and Heat Load Index (HLI), by reducing dataset to only a single annual visit or by distance to roads and trails.

**Acknowledgements:**

This work was funded by the National Park Service Cooperative Research and Training programs grant # P19AC00991. Fieldwork was completed with support from the Virginia Department of Game and Inland Fisheries, through an Endangered Species Grant from the U.S. Fish and Wildlife Service, from the National Park Service Climate Change Response Program, and from the U.S. Geological Survey (USGS) Amphibian Research and Monitoring Initiative through the Ecosystems Mission Area. This manuscript is contribution # 871 of the Amphibian Research and Monitoring Initiative (ARMI) of the USGS. Any use of trade, firm, or product names is for descriptive purposes only and does not imply endorsement by the U.S. Government.
